# Paclitaxel induces lymphatic endothelial cells autophagy to promote metastasis

**DOI:** 10.1038/s41419-019-2181-1

**Published:** 2019-12-20

**Authors:** Audrey Zamora, Melinda Alves, Charlotte Chollet, Nicole Therville, Tiffany Fougeray, Florence Tatin, Camille Franchet, Anne Gomez-Brouchet, Charlotte Vaysse, Laurent O. Martinez, Souad Najib, Julie Guillermet-Guibert, Eric Lacazette, Anne-Catherine Prats, Barbara Garmy-Susini

**Affiliations:** 1 UMR1048-I2MC, Université de Toulouse, Inserm, UT3, Toulouse, France; 2 Department of Gynecology Surgery, University Hospital Centre—Toulouse, IUCT-Oncopole, Toulouse, France; 3 UMR 1037-CRCT, Inserm, Université de Toulouse, UT3, Toulouse, France; 4 INRA Toxalim, UT3, Toulouse, France; 5 UMR 5089-IPBS, CNRS, UPS, Toulouse, France; 6 Department of Pathology, IUCT-Oncopole, Toulouse, France

**Keywords:** Breast cancer, Autophagy, Cancer

## Abstract

Cytotoxic therapy for breast cancer inhibits the growth of primary tumors, but promotes metastasis to the sentinel lymph nodes through the lymphatic system. However, the effect of first-line chemotherapy on the lymphatic endothelium has been poorly investigated. In this study, we determined that paclitaxel, the anti-cancer drug approved for the treatment of metastatic or locally advanced breast cancer, induces lymphatic endothelial cell (LEC) autophagy to increase metastases. While paclitaxel treatment was largely efficacious in inhibiting LEC adhesion, it had no effect on cell survival. Paclitaxel inhibited LEC migration and branch point formation by inducing an autophagy mechanism independent of Akt phosphorylation. In vivo, paclitaxel mediated a higher permeability of lymphatic endothelium to tumor cells and this effect was reversed by chloroquine, an autophagy-lysosome inhibitor. Despite a strong effect on reducing tumor size, paclitaxel significantly increased metastasis to the sentinel lymph nodes. This effect was restricted to a lymphatic dissemination, as chemotherapy did not affect the blood endothelium. Taken together, our findings suggest that the lymphatic system resists to chemotherapy through an autophagy mechanism to promote malignant progression and metastatic lesions. This study paves the way for new combinative therapies aimed at reducing the number of metastases.

## Introduction

Breast cancer remains the leading cause of cancer mortality worldwide, despite a significant decline in death rates due to early detection. The majority of cancer mortalities are due to the metastasis of tumor cells to other organs. Despite well-established benefits of chemotherapy on tumor growth, metastasis remains the major risk of death from this disease. Importantly, recent evidence revealed that paclitaxel, first-line chemotherapy for breast and lung cancer, increases the process of intravasation of tumor cells into the blood and lymphatic vasculature, in addition to killing tumor cells^1^. It enhances liver metastasis in murine model of breast cancer by acting directly on tumor cell invasion or by activating their immune microenvironment^2,3^.

For many carcinomas, dissemination of tumor cells via lymphatic system is the most common metastatic route. Lymphatic vessels encircle solid tumors and enhance metastasis by improving the capillary high permeability and the collecting vessels dilatation^4,5^. Nonetheless, very little is known regarding the molecular mechanisms governing cancer invasion into the lymphatic system in response to chemotherapy.

The lymphatic system comprises a network of blind-ended, thin walled lymphatic capillaries and collecting vessels. The main function of the lymphatic vasculature is to return fluid, fat, macromolecules and cells, such as leukocytes, back to the circulating blood through the lymphatico–venous junctions in the jugular area^6^. Lymphangiogenesis, the growth of new lymphatic vessels, is induced by lymphangiogenic growth factors VEGF-C and VEGF-D that can bind their receptors on lymphatic endothelial cells VEGFR2 and VEGFR-3^7,8^. It is crucially involved in the pathological conditions such as tumor metastasis, lymphedema, and various inflammatory diseases^9^. Increased expression of the lymphangiogenic factors VEGF-C and VEGF-D in tumors closely correlates with increased incidence of regional lymph node metastases in both humans and animals. VEGF-C-mediated signaling stimulates lymphatic endothelial cell (LEC) invasion and survival during lymphangiogenesis, as VEGFR-3 activates PI3K/Akt pathway^10^.

Recent studies demonstrate that chemotherapy induces lymphatic function disorders^11^. Lymph node dissection, radiation therapy, and the use of taxane were significant risk factors for lymphedema. Also, paclitaxel directly alters the VEGF-C/VEGFR-3 signaling^3^. Despite these studies suggesting an effect of chemotherapy on the lymphatic system, the mechanistic is still poorly described. Paclitaxel is one of the first-line therapy in various cancers, including breast cancer^12^; however, toxicity, resistance, and treatment failure limit its clinical use^13^. Also, recent evidence suggests that cytotoxic therapy may promote drug resistance and metastasis while inhibiting the growth of primary tumors. In that context, the widely used paclitaxel has been described to promote breast cancer metastases to the lymph nodes^14^ and it has been proposed to combine paclitaxel with anti-angiogenic therapy to reduce metastases^15^. Paclitaxel is a widely used anti-cancer drug with a well-defined mechanism of action in normal and transformed epithelial cells. However, its effect on endothelial cells is largely unknown. The emergence of drug resistance is a major limitation of the clinically success of chemotherapies. Evidence has shown that paclitaxel resistance is a process with multifactorial participation that may originate from a series of modifications including autophagy.

Autophagy is a cytoprotective function that also leads to one of the forms of cell death, by which cytoplasmic cargo sequestered inside double-membrane vesicles is delivered to the lysosome for degradation^16,17^. This process not only discards intracellular damaged organelles and misfolded or long-lived proteins, but also recycles them to provide nutrients and energy to cells exposed to various stresses. Thus, the predominant role of autophagy is considered to confer a cytoprotective function to maintain cell survival^16^. A number of studies have revealed that autophagy, which has been found to protect cancer cells from anti-cancer drug-induced death, may contribute to the development of drug resistance^18^.

Recently, many groups have independently reported that the autophagic response in blood endothelial cells is regulated by shear stress. Autophagy in endothelial cells is stimulated by cardiovascular risk factors and works as an adaptative response mediating cardiovascular protective effects^19^. In contrast, the autophagy mechanisms of endothelial cells in a tumor context have been poorly investigated. Importantly, nothing is known about the autophagic response of lymphatic endothelial cells. Here, we demonstrate that paclitaxel promotes autophagy of LECs. We have shown that paclitaxel interferes with lymphatic vessel recruitment and activation in tumors to promote metastases. Our data suggest that chemotherapy induces LECs autophagy to induce vessel survival and to stimulate metastases while tumor growth is inhibited.

## Materials and methods

### Reagents

Rabbit anti-mouse lyve-1 antibody (RDI-103PA50) was from Research Diagnostics Incorporated (Concord, MA). Donkey anti-rabbit and rat IgGs conjugated with alexa 488, 594 were from TebuBio (TebuBio, Le Perray en Yvelines, France). Anti-pSer473Akt, anti-Akt, anti-pErk, anti-Erk1/2 are from Cell Signalling Technology. Anti-Phalloidin was from Cytoskeleton (actin-stain phalloidin 488, cat PHDG1). Anti-phospho-VEGFR-3 is from Cell applications Inc. Rabbit anti-human LC3B, anti-human Notch/NCID and anti-p62 were from Cell Signaling (#2775), Goat anti-human VE-Cadherin from Santa-Cruz ((C19):SC6458) and Rabbit anti-human ATG5 and ATG7 from Sigma. Anti-human active beta-catenin was from Milliopore.

### Small-interference RNAs

Human ATG5 SMART Pool siRNA (E-004374) and human ATG7 SMART Pool siRNA (E-020112) and Non-targeting pool control (D-001910) were from Dharmacon. Human dermal lymphatic endothelial cell (HDLEC) were transfected with 30 nM and 1 nM of ATG5 and ATG7 siRNA, respectively, using Lipofectamine 2000 (Invitrogen) according to the manufacter’s recommendations. Cells were incubated at 37 °C for 48 h before experimentation.

### Cell culture

HDLECs (Promocell, Heidelberg, Germany) were cultured in endothelial growth medium MV2 (EGM-MV2) containing 5% FBS (Promocell). Proliferation, migration, branching assays were performed as previously described^20^. Positive control consists in 5% FBS, negative control consists in 0.5% serum. HDLEC cells were pre-treated with CQ (Chloroquine diphosphate salt, Sigma-C6628, 10 μM) for 1 h, after washing with phosphate-buffered saline (PBS) the cells were treated with or without PTX (Sigma- T4702, 10 nM) for 30 min, 1, 4, and 24 h.

### Mouse model of skin flap

Animal experiments were conducted in accordance with recommendations of the European Convention for the Protection of Vertebrate Animals used for experimentation. All animal experiments were performed according to the INSERM IACUC guidelines for laboratory animals' husbandry and have been approved by the local branch Inserm Rangueil-Purpan of the Midi-Pyrénées ethics committee. Skin flap was established in the left upper limbs of 6 weeks old C57Bl/6 female mice (*n* = 10). Surgically induced dermal lymphatic dissection in mice was spontaneous regenerated across skin flap incisions after 4 weeks.

### Tumor studies

To test the effect of PTX in an experimental lymph node tumor metastasis, mice (*n* = 10) were injected IP with PTX (10 mg/kg or chloroquine (50 mg/kg) 24 h before intralymphatic tumor cells injection. Anesthetized mice were inoculated with 10^6^ CMTMR 4T1 cells in a volume of 50 μL saline by intradermal injection in the left footpad, with gentle massage of the footpad. Mice were sacrificed 1 day after cell injection. Bilateral inguinal lymph nodes were removed from all mice and embedded in OCT for cryosectioning and immunofluorescence. Red fluorescent tumor cells and Lyve-1 + pixels were quantified on five different fields per lymph node cryosection.

To study orthotopic model of breast carcinoma, 50.000 syngeneic Balb/c 4T1 cells were injected in the fourth mammary fatpad. Mice (*n* = 7) were treated IP with PTX (10 mg/kG) or chloroquine (50 mg/kg) 3, 6, 9, and 12 days after tumor injection. Animals were sacrificed after 14 days, tumor and inguinal lymph nodes were excised and embedded in OCT.

### Whole-mount immunostaining

For whole-mount immunostaining, samples were fixed in 4% formaldehyde in PBS for 2 h on ice. After washing twice in PBS, sample were permeabilized in PBS 0.1% TritonX-100 (PBST) and saturated for 2 h in PBS containing 0.1% TritonX-100, 3% milk (PBSMT) at room temperature (RT). After washing twice in PBS, samples were incubated with primary antibodies: anti-mouse podoplanin from developmental studies hybridoma bank at 0.3 μg/mL; rabbit anti-mouse Lyve-1 (Interchim 70R-LR005) at 2 μg/mL and rat anti-mouse CD31 (BD 553370) at 2.5 μg/mL in PBST overnight at 4 °C with gentle agitation. Samples were washed every 15 min during 3 h in PBST and incubated overnight at 4 °C with secondary antibodies in PBST. Sample were then washed as described for the primary antibodies and mounted in Mowiol (Mowiol 4–88, Hoechst) supplemented with 2.5% anti-bleaching agent DABCO (Sigma-Aldrich, France). Images were recorded using an LSM780 laser scanning confocal microscope (Zeiss). Negative controls were performed with no primary antibody.

### Statistical analysis

All statistical analyses were performed using either a two-tailed Student's *t*-test for analyses of two groups, or one-way analysis of variance (ANOVA) for analyses of three or more groups. One-way ANOVA was followed by post-hoc test of Bonferroni. All experiments were performed three times, where the quantification is reported as the average + /− SEM of three separate animal experiments.

### Immunocytochemistry

Cells were cultured as a confluent monolayer on coverslip, treated as described, washed with PBS and fixed with 4% paraformaldehyde for 10 min. Subsequently, the cells were permeabilized with 0.3% Triton for 1 min, and washed with PBS. ECs were incubated for 5 min in blocking buffer (1% bovine serum albumin (BSA)-2% fetal bovine serum (FBS) in PBS) before exposure to rabbit anti-LC3 antibody (1:200) overnight at 4 °C. Cells were then incubated with anti-rabbit antibody at 7.5 μg/mL for 1 h. Goat anti-VE-cadherin was used at 1 μg/mL and anti-goat antibody at 4.6 μg/mL. For actin staining phalloidin was diluted 1:200. Nuclei were counterstained with 4-6-diamidino-2-phenylindole (DAPI).

### Image analysis

The number of LC3 punctae positives cells was assessed under a fluorescence microscope (Leica DMi8) at 40x objective lens magnification, from at least 15 fields. Representative images of LC3B quantification were taken on a confocal microscope (LSM780) at 63x objective magnification. Maximum intensity projection images were exported from Zen software as full-resolution tagged image file format (TIFF) images and opened using Photoshop CS6 software (Adobe Systems, Inc., San Jose, CA). Gaps quantification were done manually using Image J software (http://imagej.nih.gov/ij/; provided in the public domain by the National Institutes of Health, Bethesda, MD, USA), from five random fields using a 40x objective.

## Results

### Paclitaxel inhibits lymphatic healing

The lymphatic system, acting in concert with the blood vascular system, is maintaining tissue homeostasis. The taxane chemotherapeutic agents are most often associated with hypersensitivity reactions, encountered either during or shortly after infusion, leading to edema^21–23^. With current treatment strategies aimed at multimodal protocols, including surgery, radiation, and chemotherapeutic regimens, it is not surprising that wound healing becomes a matter of critical importance.

**Fig. 1:**
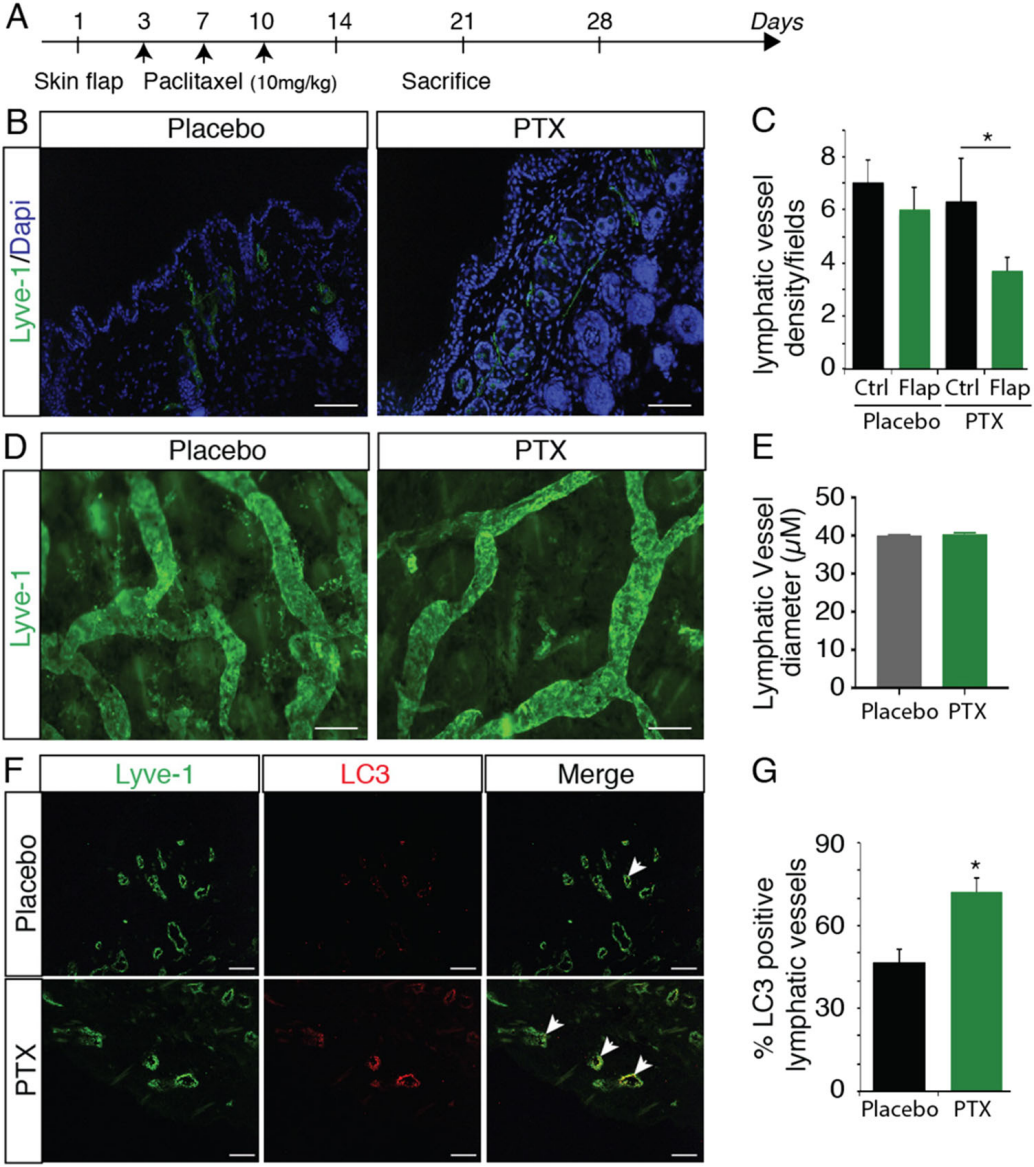
PTX reduces lymphatic healing. **a** Schematic representation of the skin flap experimental procedure. **b** Representative fluorescent images of Lyve-1-positive regenerated lymphatic vessels from PTX-treated mice 4 weeks after skin flap. Scale bar: 50 μm. **c** Quantification of the lymphatic vessel density in PTX-treated mice, *p < 0.05. **d** Immunostaining for Lyve-1 shows no difference in lymphatic vessel size in PTX-treated mice. Scale bar: 200 μm. **e** Quantification of the lymphatic vessel diameter in PTX-treated mice skin. **f** Immunostaining for Lyve-1 (green) and LC3 (red) shows increase of LC3 expression in lymphatic vessels from PTX-treated mice. Scale bar: 50 μm. **g** Quantification of the percentage of LC3-positive lymphatic vessels in PTX-treated mice skin.

To study the effect of paclitaxel (PTX) on lymphatic system in vivo, we first performed a pectoral skin flap model to examine wound-healing lymphangiogenesis (Fig. 1). Mice were treated with three intraperitoneal injections of paclitaxel and dermal lymphatic and blood vasculature were analyzed 4 weeks after surgery (Fig. 1a). We observed a delay in lymphangiogenesis in the scar area from PTX-treated mice compared to adjuvant-treated mice (Fig. 1b, c). Surprisingly, paclitaxel, had no effect on lymphatic and blood vessel shape and diameter (Fig. 1d, e and Supplementary Fig. 1), but we observed that the inhibition of lymphangiogenesis was correlated with an increase of the marker of autophagy LC3 expression in lymphatic endothelial cells (Fig. 1f, g).

### PTX inhibits lymphatic endothelial cells (LEC) functions in vitro

**Fig. 2:**
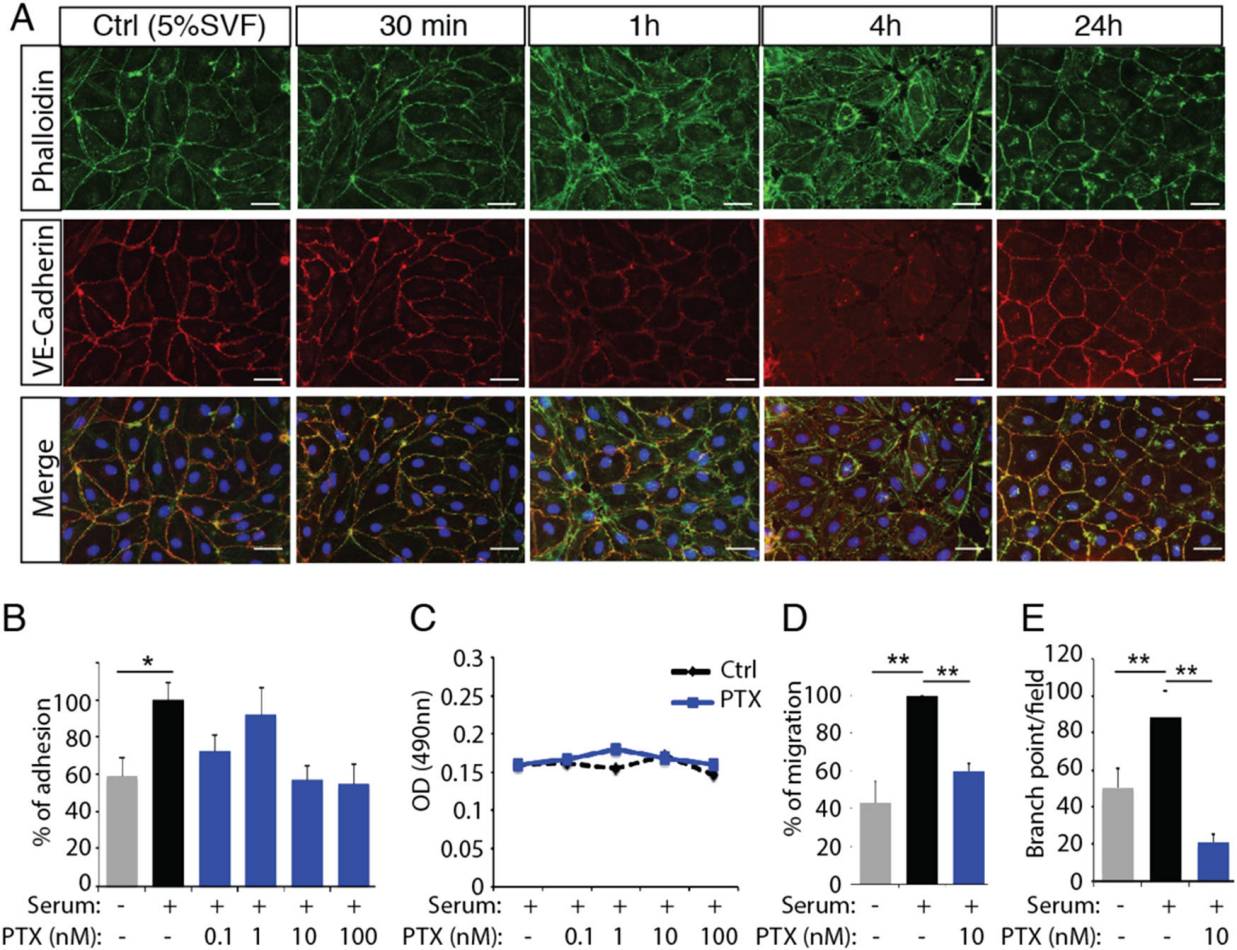
PTX inhibits lymphatic endothelial cell function in vitro. **a** Phalloidin (green) and VE-cadherin (red) immunostaining on HDLEC stimulated by PTX (10 nM) for 30 min, 1 h, 4 h, and 24 h shows a downregulation of VE-cadherin expression after 1 h and 4 h associated with an alteration of cortical actin organization at cellular junctions as show with phalloidin immunostaining. Scale bar 50 μm. **b**, **c** Increasing doses of PTX (0.1–100 nM) reduced HDLEC adhesion in the presence of 5% SVF without significant effect on HDLEC survival as shown by MTT assay. **d** Quantification of the percentage of HDLEC migration using scratch wound healing assay shows an inhibition of serum-induced (5%SVF) migration by PTX. **p < 0.01. **e** PTX antagonizes serum-induced (5%SVF) HDLEC branching in matrigel. **p < 0.01.

To determine how paclitaxel modulates the lymphatic system, we tested its effect in vitro on human dermal lymphatic endothelial cells (HDLEC) (Fig. 2). Cortical actin was found to be organized in a jagged fashion 1 h post-exposure with PTX compared to untreated cells, which displayed a linear distribution. Alteration of cortical actin preceded retraction of the cellular membranes observed after 4 h of PTX treatment (Fig. 2a) and was accompanied by a marked decrease of membrane-distributed VE-cadherin, which no longer co-localized with cortical actin. However, after 24 h of incubation of the HDLEC with PTX, linear organization of cortical actin and continuous VE-cadherin staining along the cell border were recovered. PTX-induced actin cytoskeleton polymerization was associated with decrease in LEC adhesion (Fig. 2b) without any effect on cell survival (Fig. 2c). In parallel, we found that PTX inhibited HDLEC migration and tube formation in the presence of 5% serum (Fig. 2d, e).

### PTX induces autophagy in lymphatic endothelial cells

**Fig. 3:**
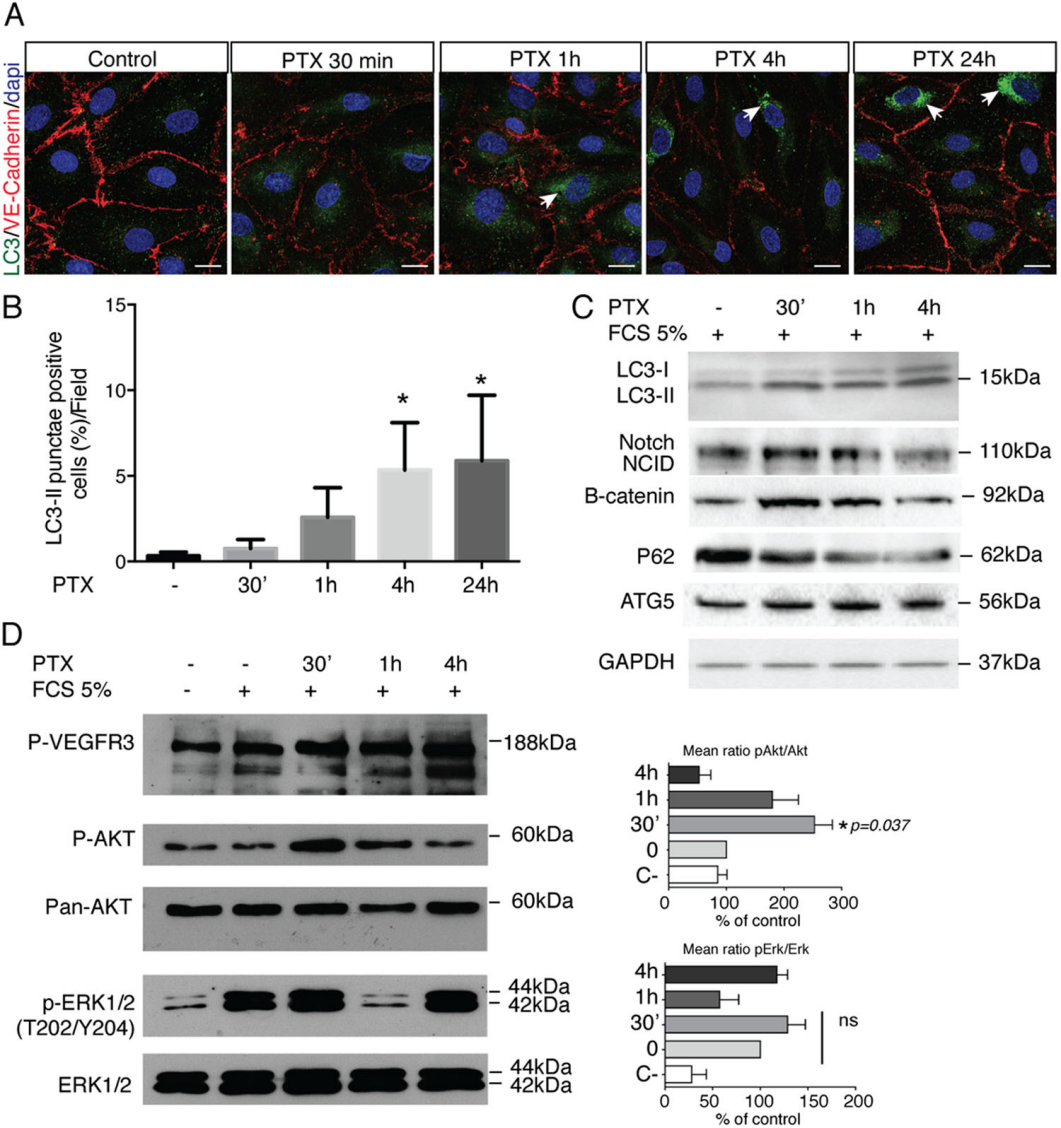
PTX induces HDLEC autophagy. **a** LC3 (green) and VE-cadherin (red) immunostaining on HDLEC stimulated by PTX (10 μM) for 30 min, 1 h, 4 h, and 24 h shows an upregualtion of LC3 dots after 1 h, 4 h and 24 h of stimulation. **b** Quantification of the LC3-positive HDLEC treated with PTX. *p < 0.05. **c** Immunoblot analysis of LC3-I, LC3-II, Notch/NCID, beta-catenin, P62 and ATG5 in HDLECs stimulated by PTX (10 nM) for 30 min, 1 h, and 4 h. **d** Immunoblot analysis of P-VEGFR-3, pS473Akt, Akt, pERK, and ERK1/2 in HDLECs stimulated by PTX (10 nM) for 30 min, 1 h, and 4 h.

We next analyzed the effect of paclitaxel on HDLEC autophagy. Microtubule-associated protein 1 light chain 3A/B (LC3A/B) was used as a marker of autophagy. LC3 is a soluble protein that is proteolytically modified by a C-terminal cleavage to generate a form (LC3-I) that is subsequently conjugated to phosphatidylethanolamine (PE) to produce LC3–II, which is recruited to phagophore membranes. Meanwhile, the high conversion of LC3-I into LC3-II reflects either a high-autophagic flux, or a blockade in autolysosomal degradation^24^. The ratio of LC3-II/GAPDH was higher in HDLECs treated for 30 min to 4 h with PTX (10 nM), suggesting that paclitaxel induces autophagy in HDLECs (Fig. 3a–c). To confirm the HDLEC autophagy, we show that a key regulator of autophagy, the autophagy-related protein 5 (ATG5), was upregulated in PTX-stimulated HDLECs whereas P62 level is altered (Fig. 3c). Notch pathways did not seem to be modified by PTX treatment (Fig. 3c). However, active beta-catenin was upregulated after 30 min and 1 h of PTX stimulation, but decreased after 4 h, suggesting a decrease of cell adhesion at this time point (Fig. 3c). Conversely, the increase of autophagy markers ATG5 and LC3-II was associated with a transient dephosphorylation of VEGFR-3 (Fig. 3d), in line with the decrease of cell migration and tube formation observed in Fig. 2d, e. The Akt phosphorylation was activated after 30 min, but significantly decreased after 4 h treatment with PTX following the beta-catenin activation. In contrast, Erk phosphorylation was not significantly modified by PTX in LEC (Fig. 3d).

**Fig. 4:**
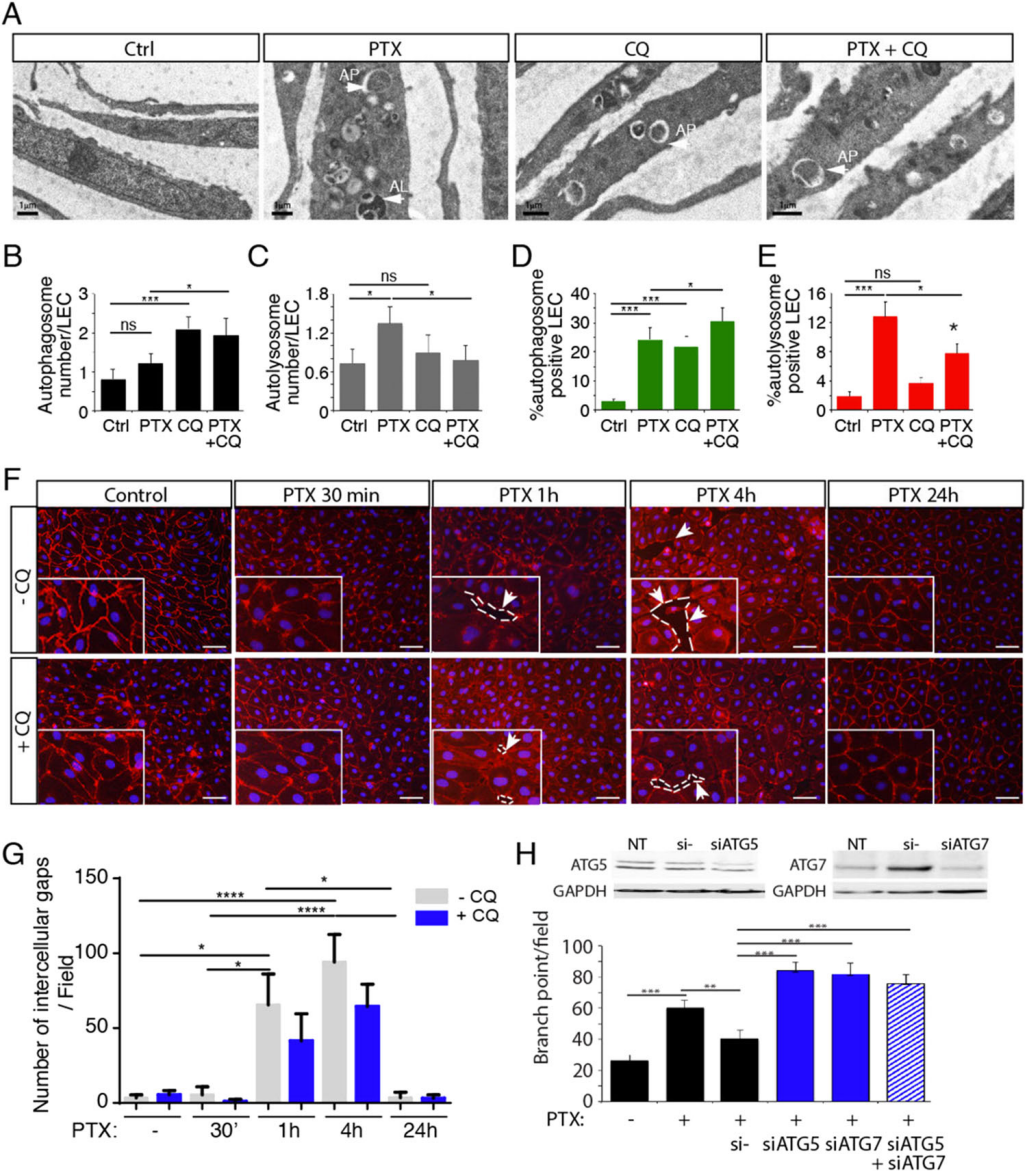
PTX-induced gap formation in HDLEC monolayer is reversed by Chloroquine. **a** Transmission electron microscopy representative images of PTX, CQ, PTX + CQ-treated LEC. **b** Quantification of the lymphatic endothelial autophagosome. *p < 0.05; ***p < 0.001. **c** Quantification of the lymphatic endothelial autolysosomes. *p < 0.05; ***p < 0.001. **d**, **e** Quantification of autophagosomes (**d**) and autolysosomes (**e**) in PTX and CQ-treated LEC using mRFP-GFP-LC3 assay. **f** VE-cadherin immunostaining on HDLEC stimulated by PTX (10 nM) for 30 min, 1 h, 4 h, and 24 h shows gap between endothelial cells after 1 h and 4 h that is in part prevented by chloroquine (CQ). Scale bar 50 μm. **g** Quantification of the lymphatic endothelial gap formation. *p < 0.05; ***p < 0.001.; ****p < 0.0001. **h** Knockdown of ATG5 and ATG7 reversed PTX inhibition of HDLEC branching in matrigel. **p < 0.01; ***p < 0.001.

To confirm the induction of autophagy in LECs, Transmission Electron microscopy (TEM) has been performed to better detect autophagy/autophagosomes in PTX-treated LEC (Fig. 4a–c). Autophagosome and autolysosome numbers have been quantified in PTX, CQ, and CQ + PTX-treated LEC. As expected, we observed that CQ, known to inhibit the fusion between autophagosomes and autolysosomes, increases autophagosome number (Fig. 4b). PTX induced an increase of autolysosome formation that was reversed by CQ treatment (Fig. 4c), which demonstrate that PTX induces autophagy in LECs.

Finally, autophagy flux was measured by performing mRFP-GFP-LC3 assay on LECs treated by PTX and CQ (Fig. 4d, e and Supplementary Fig 2). We observed an increase of autophagosome formation after PTX and CQ treatment and an increase of autolysosome formation after PTX treatment that was reversed by CQ (Fig. 4d, e).

### PTX induced disruption of the endothelial barrier

Barrier function is largely determined by structural and junctional proteins. To assess the effect of PTX on endothelial barrier integrity, we analyzed the distribution of VE-cadherin in a confluent monolayer of HDLEC. Cell retraction with the concomitant loss of VE-cadherin from the cell surface were associated with a significant increase of intercellular gaps (Fig. 4f, g). In the regions of gap formation slight traces of VE-cadherin were still visible at some remaining cell-cell contacts (Fig. 4f). Gaps numbers appeared to be also significantly reduced 24 h following incubation with PTX. Furthermore, when PTX-treated cells were pre-treated with CQ, VE-cadherin staining was preserved and the formation of the gaps was limited (Fig. 4f, g), suggesting that autophagy could function as a degradation mechanism for this protein. Then, we studied the effect of siRNA-mediated knockdown of ATG5 knockdown on the LEC monolayer. We observed a delay in the gap formation induced by PTX in comparison with non-targeting control siRNA with a complete inhibition of gap formation following PTX treatment for 1 h (Supplementary Fig 3), which confirms the effect observed with CQ (Fig. 4g).

These results indicate that the loss of interaction between adjacent endothelial cells upon stabilization of microtubule by PTX, is dependent on actin disorganization and adherent junction disassembly. Increase of intercellular gaps in PTX-treated LEC may reflect barrier dysfunction through increased vascular permeability.

### PTX enhances tumor cell invasion into the lymph nodes

**Fig. 5:**
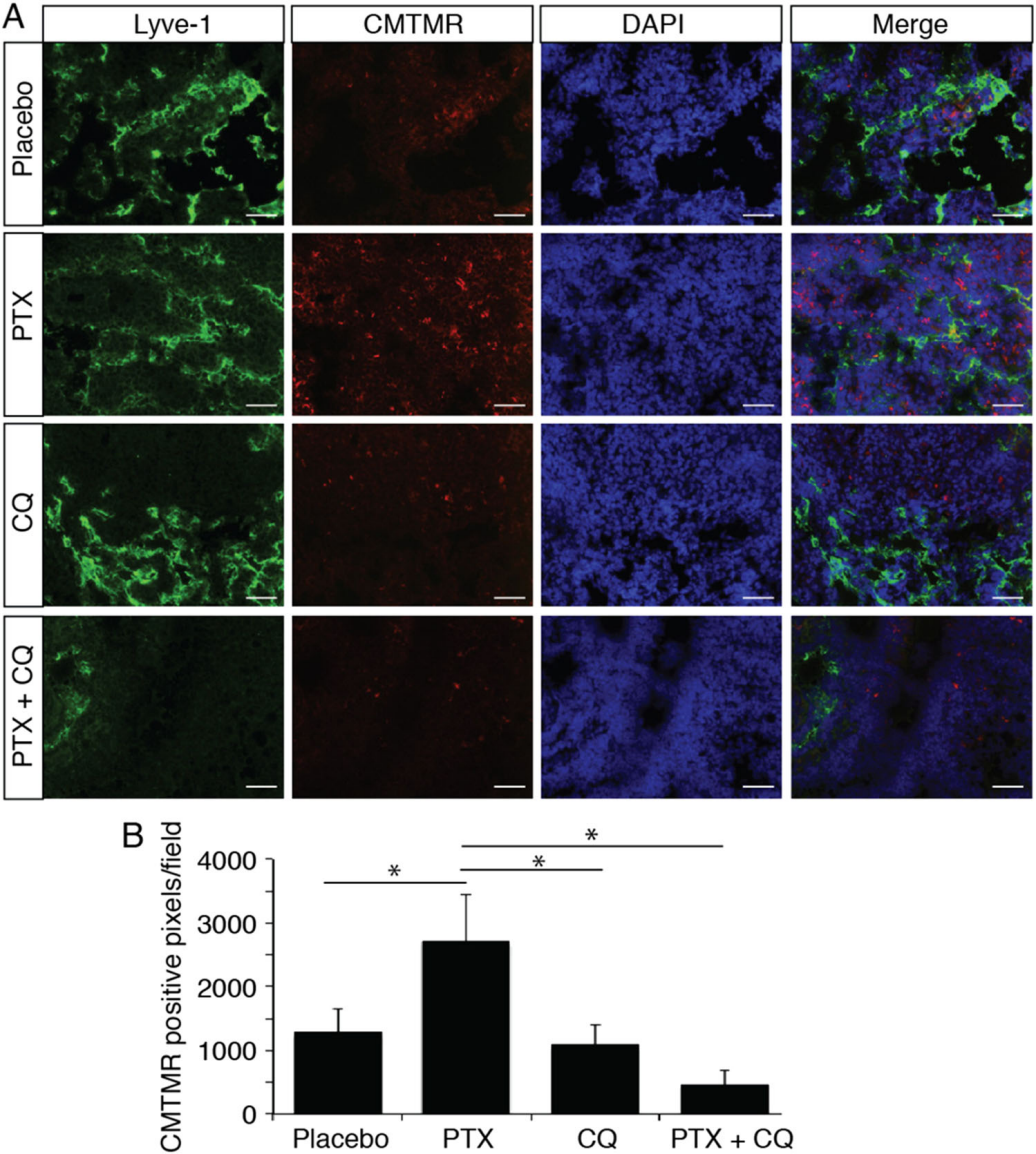
Chloroquine inhibits PTX-induced tumor cells extravasation. **a** Representative fluorescent images of “extravasated” CMTMR-labeled tumor cells (red) in the inguinal lymph node from PTX or CQ-treated mice. **b** Quantification of CMTMR-labeled tumor cells in the inguinal lymph node. *p < 0.05.

The lymphatic system serves as route for tumor metastasis to the lymph nodes. To determine whether PTX might promote tumor cells metastasis to lymph nodes, cell tracker (CMTMR) labeled 4T1 tumor cells were injected into the footpads of mice pre-treated with PTX. Treatment with PTX prior to injection into the footpad strongly suppressed tumor cell retention in lymph nodes without affecting lymphatic vessel density (Fig. 5a, b).

In contrast, inhibition of PTX-induced lymphatic autophagy by chloroquine decreased lymph node lymphatic vessel density and prevented 4T1 cell retention in vivo (Fig. 5a, b), indicating that PTX resistance of lymphatic vessels using autophagy-mediated lymphatic metastasis. Our studies suggest that PTX induced lymphatic permeability to increase the tumor cell transendothelial migration into the lymph nodes. As lymphatic physiologic role is to transport immune cells, it was tempted to postulate that an increase in lymphatic permeability could also lead to an increase of immune cells infiltration. We then investigated whether lymphocyte trafficking was modified in metastatic breast cancer patients by measuring lymphocyte infiltration in ten neoadjuvant and ten adjuvant PTX-treated patients tissue biopsies obtained from the biology resource center of Rangueil hospital (Toulouse). We observed an increased CD3-positive lymphocyte infiltration in the vicinity of tumor lymphatic vessels from patients treated with neoadjuvant therapy (Supplementary Fig. 4), supporting the hypothesis that neoadjuvant PTX therapy increases the tumor lymphatic vessels permeability.

### PTX inhibits tumor lymphangiogenesis but had no effect on angiogenesis

**Fig. 6:**
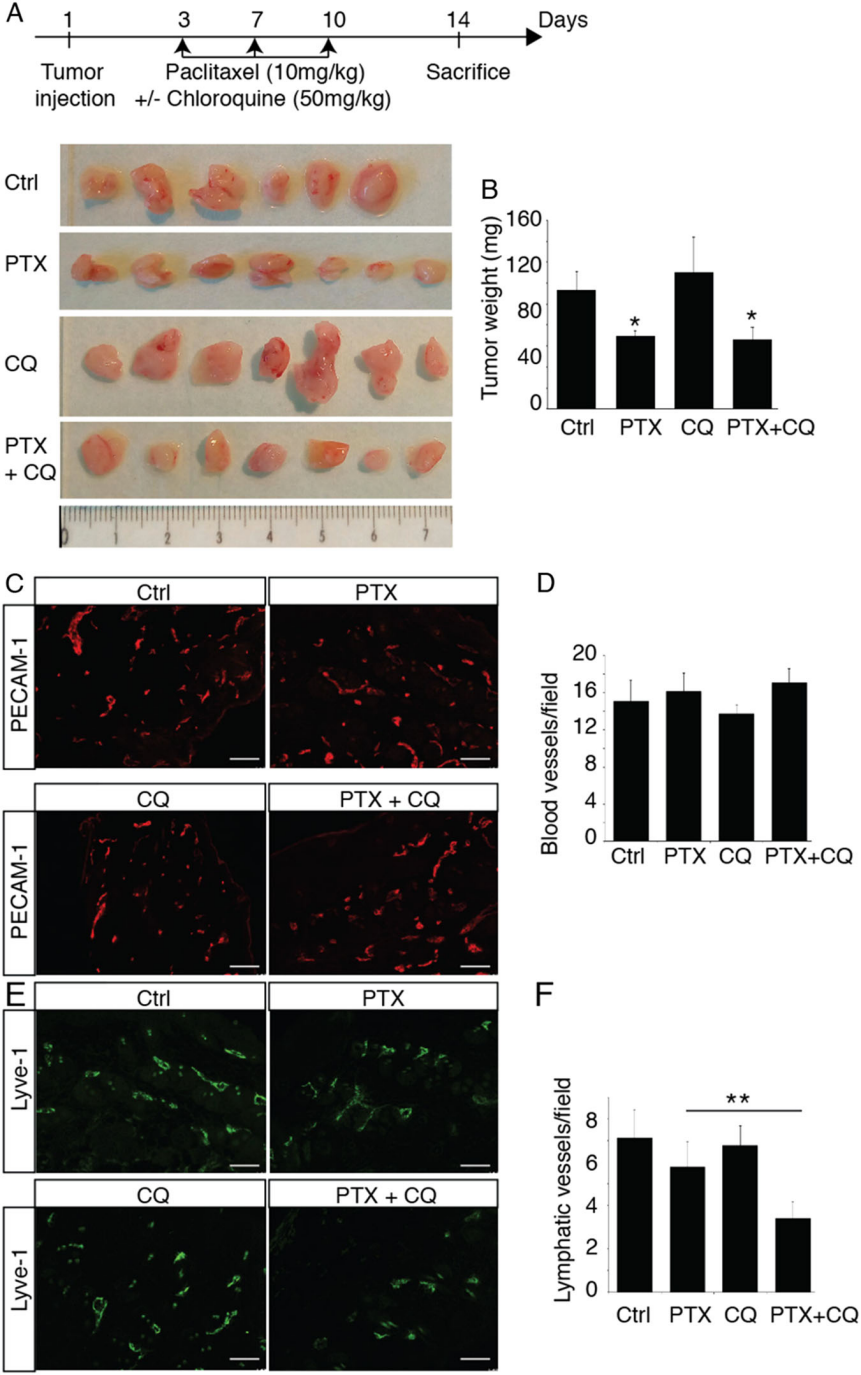
Chloroquine inhibits PTX-induced tumor lymphangiogenesis. **a** Schematic representation of the skin flap experimental procedure and images of 4T1 tumors from mice treated with PTX, CQ or PTX + CQ. **b** Quantification of tumor weights. *p < 0.05. **c** Representative fluorescent images of PECAM-1-positive blood vessels from PTX- or CQ-treated tumor bearing mice. Scale bar: 50 μm. **d** Quantification of the blood vessel density in 4T1 tumors from PTX- or CQ-treated mice. **e** Representative fluorescent images of Lyve-1-positive lymphatic vessels from PTX- or CQ-treated tumor bearing mice. Scale bar: 50 μm. **f** Quantification of the lymphatic vessel density in 4T1 tumors from PTX- or CQ-treated mice, **p < 0.001.

To study the effect of PTX on tumor lymphangiogenesis, we used chemotherapy in 4T1-bearing mice, a murine syngeneic model of metastatic breast cancer. In this model, mice exhibit reproducible inguinal lymph nodes metastases after 2 weeks. Following tumor transplantation, mice were injected IP with PTX (10 mg/kg) at days 3, 6, and 9 and tumor were harvested at day 14 (Fig. 6a). As expected, PTX reduced tumor weight (Fig. 6a, b), but had no effect on other organ weights (not shown). This effect on tumor size was attributed to the anti-mitogenic activity of chemotherapy on primary tumor as we did not observe any effect on tumor angiogenesis (Fig. 6c, d). In parallel, we observed a slight inhibition of tumor lymphangiogenesis induced by PTX that was dramatically increased by the association with chloroquine (50 mg/kg), suggesting that autophagy protects the lymphatic endothelium from the adverse effect of chemotherapy (Fig. 6e, f). These data suggest that an inhibition of lymphatic resistance to chemotherapy can be hampered by the delivery of chloroquine, an autophagy inhibitor.

### Chloroquine inhibits PTX-induced lymphatic metastasis to the lymph nodes

**Fig. 7:**
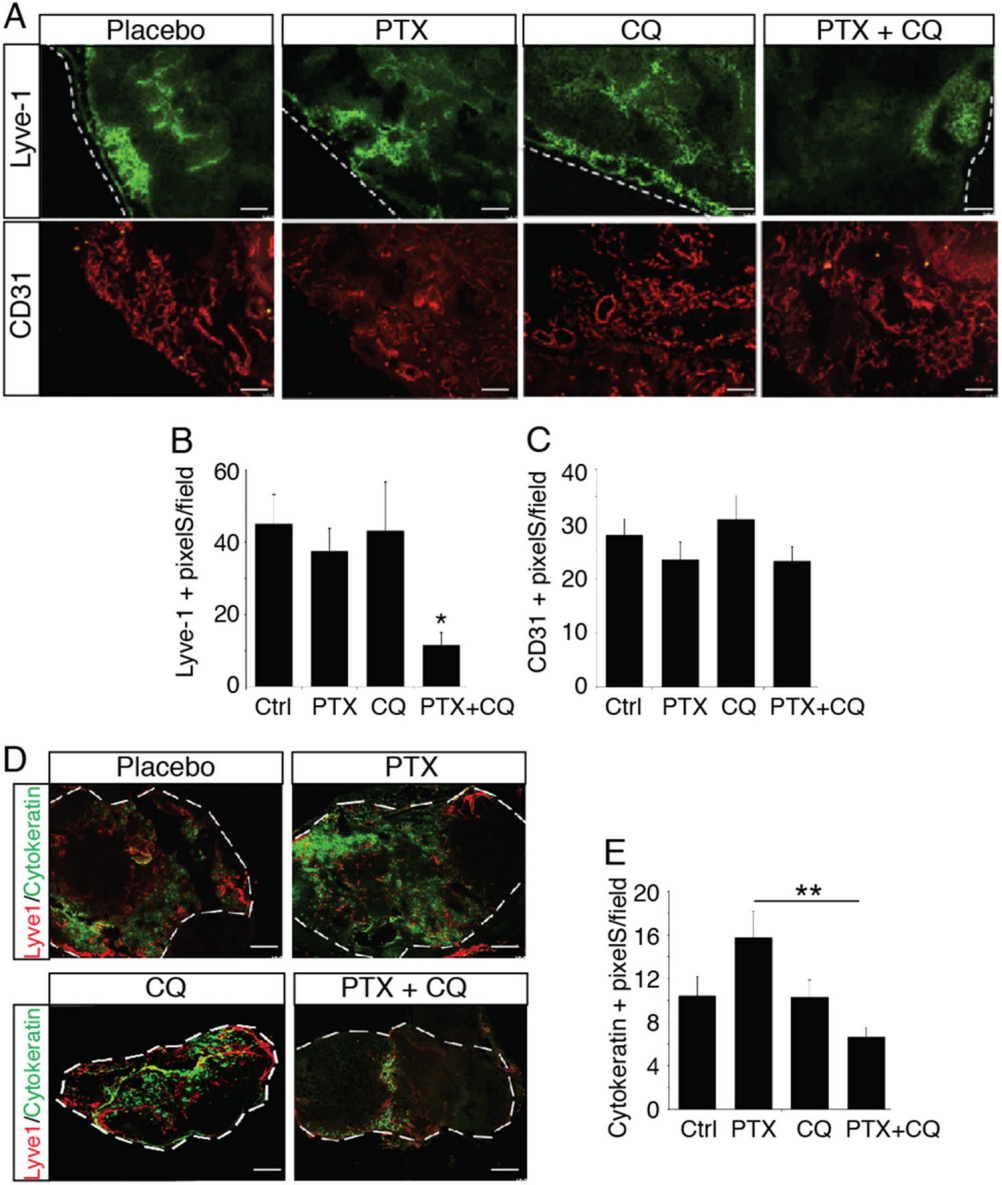
Chloroquine inhibits PTX-induced tumor metastasis. **a** Representative fluorescent images of Lyve-1-positive lymphatic vessels and PECAM-1-positive blood vessels in the inguinal lymph node from PTX- or CQ-treated tumor bearing mice. **b**, **c** Quantification of the lymphatic (**b**) and blood (**c**) vessel density in the inguinal lymph node from 4T1 tumors bearing mice treated with PTX or CQ, **p < 0.001. **d** Representative fluorescent images of Lyve-1-positive lymphatic vessels and cytokeratin-positive tumor metastases in the inguinal lymph node from PTX- or CQ-treated tumor bearing mice. **e** Quantification of the cytokeratin-positive tumor metastases in the inguinal lymph node from 4T1 tumors bearing mice treated with PTX or CQ, **p < 0.001.

To further study the effect of PTX on lymphatic metastasis, we analyzed the sentinel lymph nodes (Fig. 7). PTX and chloroquine alone had no effect on lymph node lymphangiogenesis and angiogenesis (Fig. 7a–c). In contrast, the combination of these molecules strongly reduced lymph node lymphangiogenesis and metastases to the lymph nodes and lymphoid tissues (Fig. 7d, e and Supplementary Fig. 5). This was associated with a strong upregulation of LC3 expression in lymphatic vessels from mice treated with PTX and CQ (Supplementary Fig. 6).

## Discussion

Lymphatic vessels develop in solid tumors but also in the lymph nodes to provide an easier way for metastasis dissemination due to their high permeability^10^. Thus, lymphangiogenesis appears as a major therapeutic target in treatments against cancer dissemination^25^. However, the effect of cytotoxic agents on the proliferation of the lymphatic vessels during tumor progression has been poorly understood. Here, we identify a mechanism of autophagy developed by the lymphatics in response to PTX chemotherapy to maintain routes for metastases to escape from primary tumor. We recently identified stress-induced molecular regulation of lymphangiogenic growth factors VEGF-C and VEGF-D in tumors^7,8^. We found that hypoxia strongly stimulates VEGF-C synthesis in breast cancer in both primary tumors and in metastatic loci^26^. In parallel, VEGF-D is induced by inflammatory stress in breast cancer to stimulate lymphatic vessel dilatation to increase metastases^26^. These studies revealed that lymphangiogenesis, as well as lymphatic vessel architecture are both key actors in tumor dissemination. Altogether, many targets and signaling pathways have been identified in the past decade to block the tumor lymphangiogenesis. In this study, we would like to identify whether chemotherapy could interfere with these processes. Despite its crucial role in tumor metastasis, the effect of first-line chemotherapy on the lymphatic system has surprisingly never been investigated.

In the present study, we have found that paclitaxel, a drug that induces mitotic arrest due to activation of the mitotic checkpoint, could promote lymphatic endothelial cells autophagy. Despite ample evidence showing that autophagy plays an important role on blood endothelium, its role in tumoral lymphatic vasculature remains unclear. Chloroquine, an autophagy blocker, not only reduces tumor growth but also improves normalization and function of tumor-associated blood vessels^27^. Recently, a study suggested that photodynamic therapy of cancer could promote lymphatic endothelial cells autophagy^28^. Here, we have investigated the effect of chemotherapy on the lymphatic endothelium both in vitro and in vivo to better understand whether the lymphatic vessel resistance to chemotherapy could continue to provide route for metastases.

Autophagy has been first described as negatively regulated by Akt in response to mitogens via activation of Target of Rapamycin (TOR)^29^. However, other studies revealed an mTOR-independent mechanism by which Akt can suppress autophagy^30^. Here, we report that Akt regulates autophagy in a TOR-independent manner in LEC in response to chemotherapy. Interestingly, this is associated with a dephosphorylation of VEGFR-3 and a decrease in adhesion molecule expression. The rearrangement of the actin cytoskeleton was followed by a transient lymphatic permeability increase generated by endothelial gap formation induced by PTX. This was confirmed in vivo by an experimental approach aiming at injecting tumor cells into the lymphatic system to promote metastasis independently of a primary tumor. Our studies indicate that systemic PTX might promote widespread of tumor metastases in the lymph node by modulating vessel permeability. This process was independent from lymph node lymphangiogenesis as we found that PTX strongly inhibits lymphangiogenesis only in association with chloroquine, the autophagy inhibitor. The discordance with the inhibition of lymphangiogenesis by PTX observed in the skin flap model is probably attributed to a compensation induced by the secretion of lymphangiogenic factors VEGF-C and -D by the primary tumor.

Altogether, our results suggest that PTX increased lymphatic metastases by promoting LEC autophagy to maintain lymphatic vessel structure during chemotherapy, and by inducing vessel permeability. Also, intravenous chemotherapy has poor access to metastatic lymph nodes and is limited by short-lived drug concentrations. Therefore, the administration of chemotherapy via the lymphatic network appears as a new concept for the prevention and treatment of metastatic lymph nodes. Altogether, these data reveal the need for a better understanding of the action of chemotherapies on the lymphatic vascular endothelium. In conclusion, this study allows an advance in the fundamental knowledge of the lymphangiogenic process in breast cancer metastasis. We have demonstrated the importance of the maintenance of a functional lymphatic architecture in tumorous lymphangiogenesis after chemotherapy. Data from the literature suggest that paclitaxel induces metastasis despite an inhibitory effect on tumor growth. Here, we highlight which mechanism is specifically activated in lymphatic endothelial cells to allow the tumor dissemination.

## Supplementary information

Supplemental figure 1

Supplemental figure 2

Supplemental figure 3

Supplemental figure 4

Supplemental figure 5

Supplemental figure 6

supplemental informations
