# Supplementary figures and images for: Paclitaxel induces lymphatic endothelial cells autophagy to promote metastasis
Source: Cell Death Dis. 2019 Dec 20;10(12):956. doi: 10.1038/s41419-019-2181-1 (PMC6925245; doi:10.1038/s41419-019-2181-1)

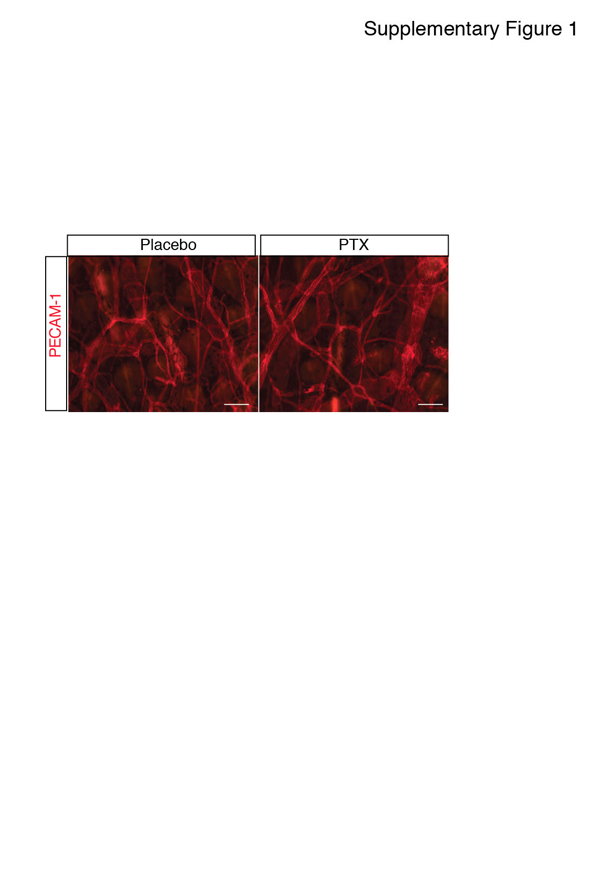

Supplement: Supplementary file 1 — Supplemental figure 1 [file 41419_2019_2181_MOESM1_ESM.tif]

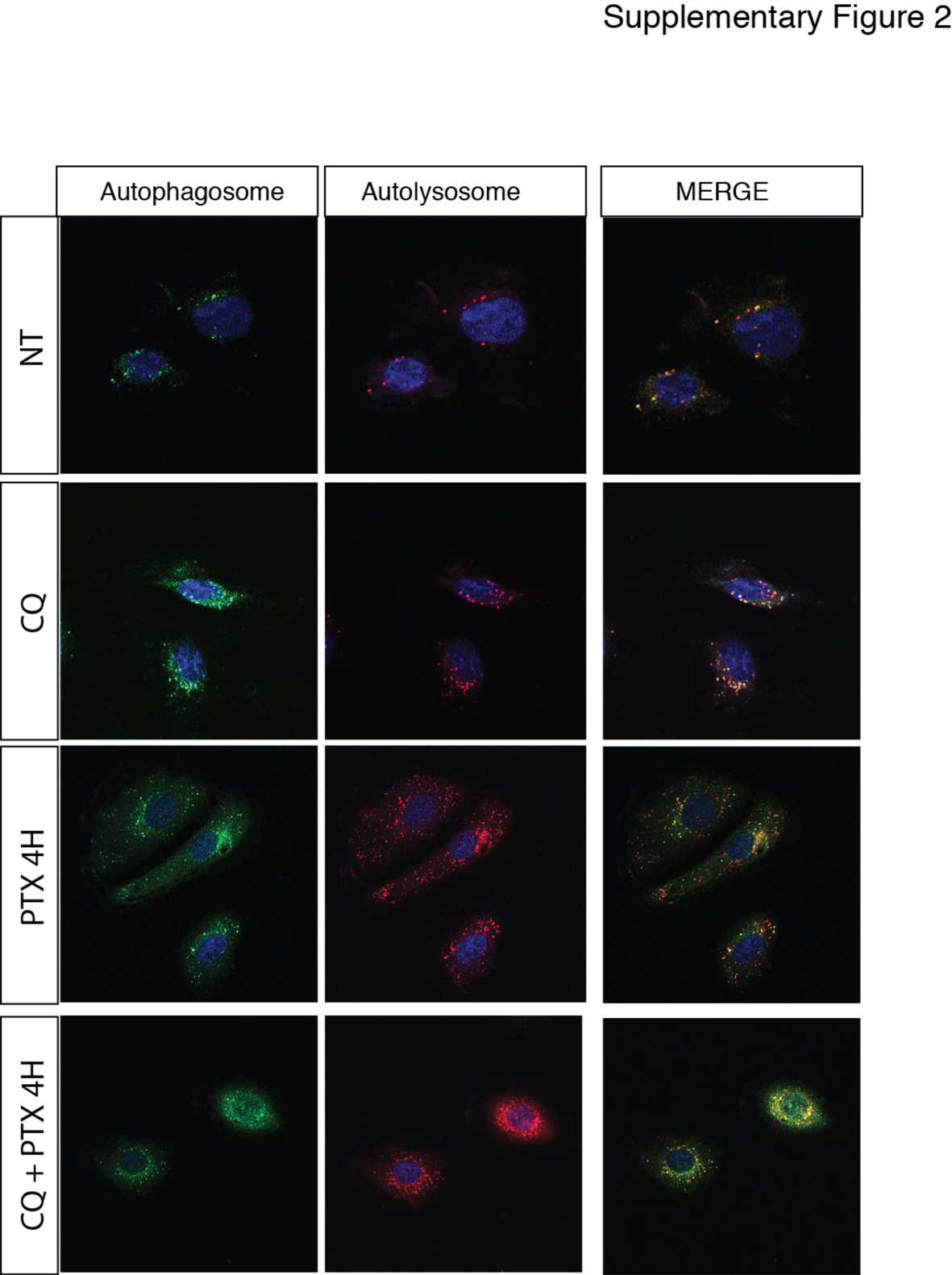

Supplement: Supplementary file 2 — Supplemental figure 2 [file 41419_2019_2181_MOESM2_ESM.tif]

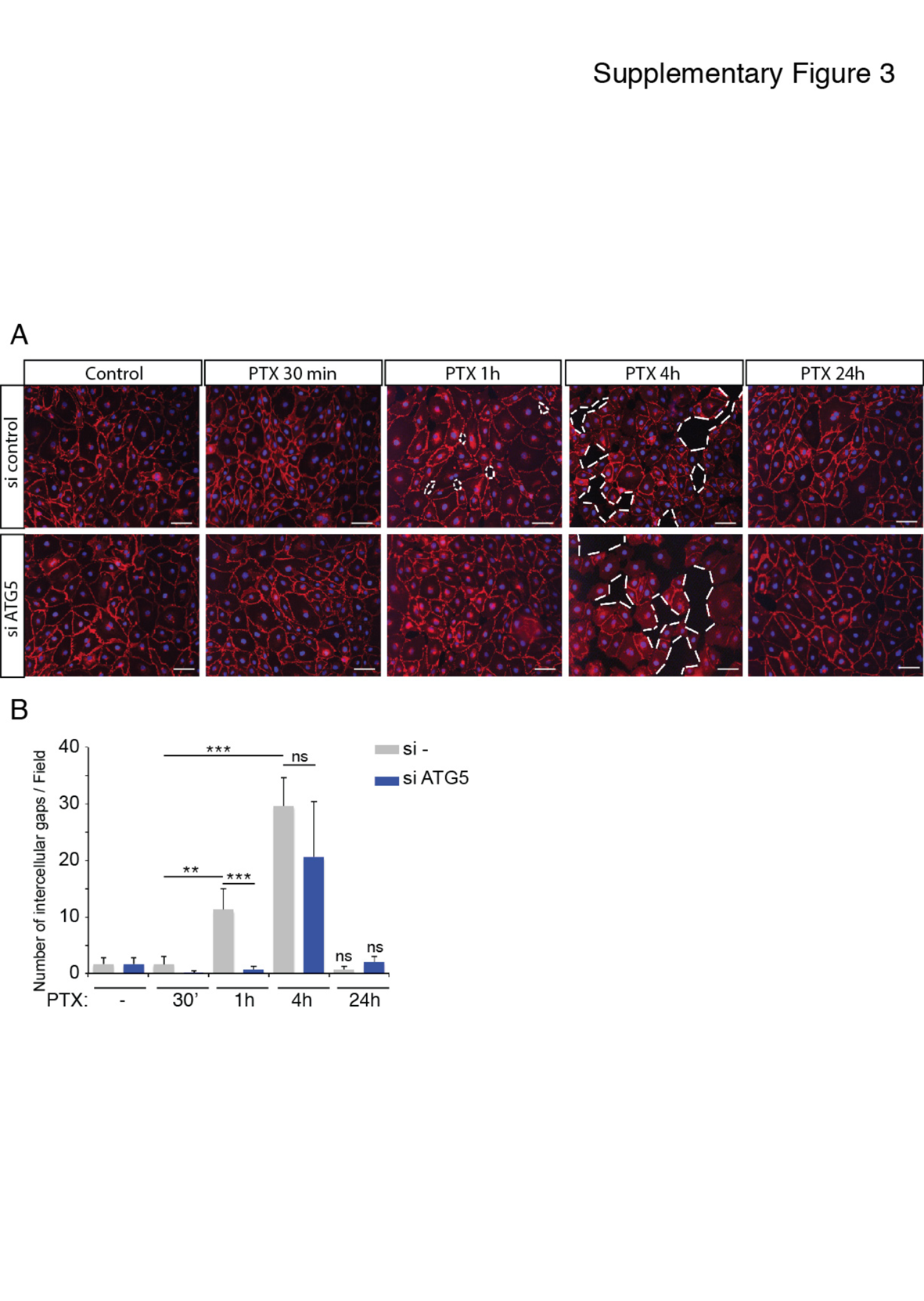

Supplement: Supplementary file 3 — Supplemental figure 3 [file 41419_2019_2181_MOESM3_ESM.tif]

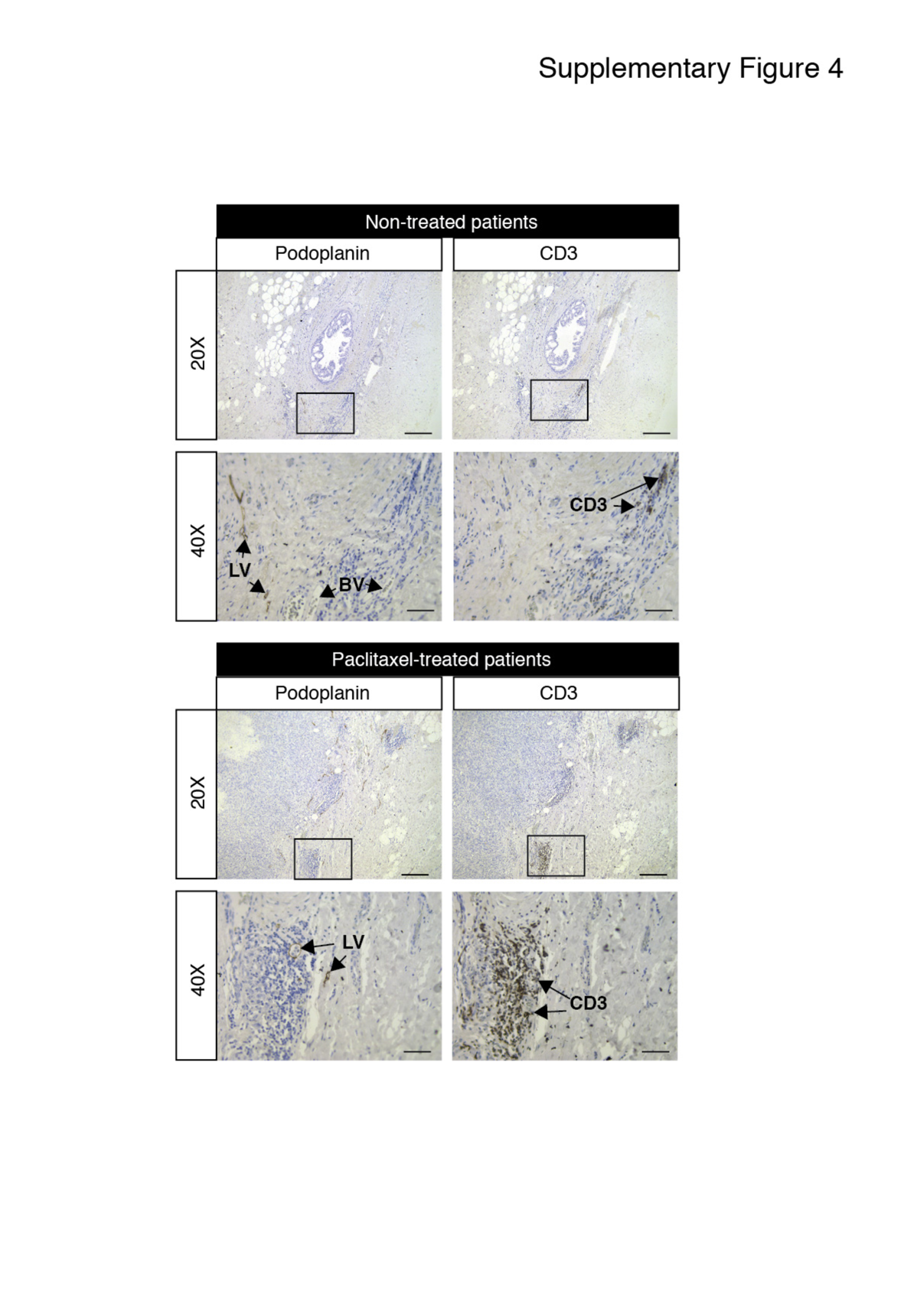

Supplement: Supplementary file 4 — Supplemental figure 4 [file 41419_2019_2181_MOESM4_ESM.tif]

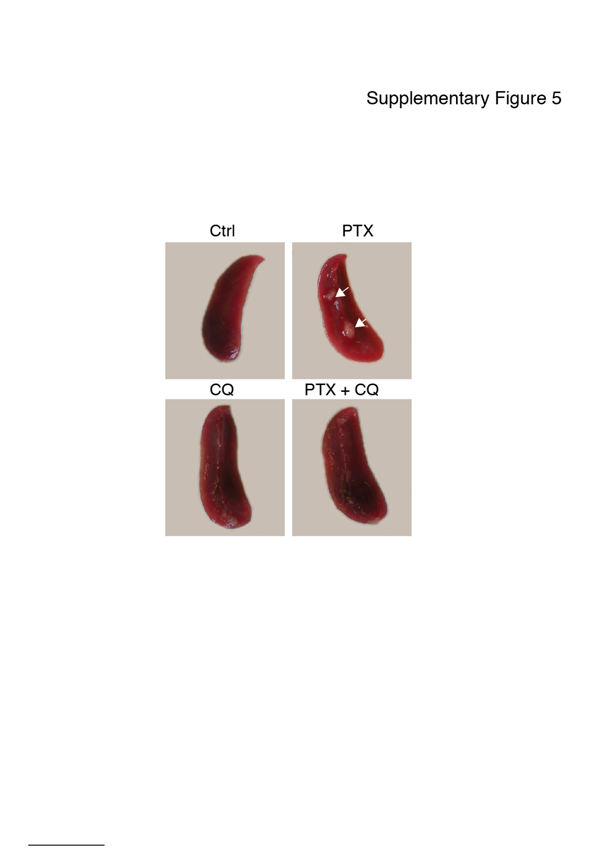

Supplement: Supplementary file 5 — Supplemental figure 5 [file 41419_2019_2181_MOESM5_ESM.tif]

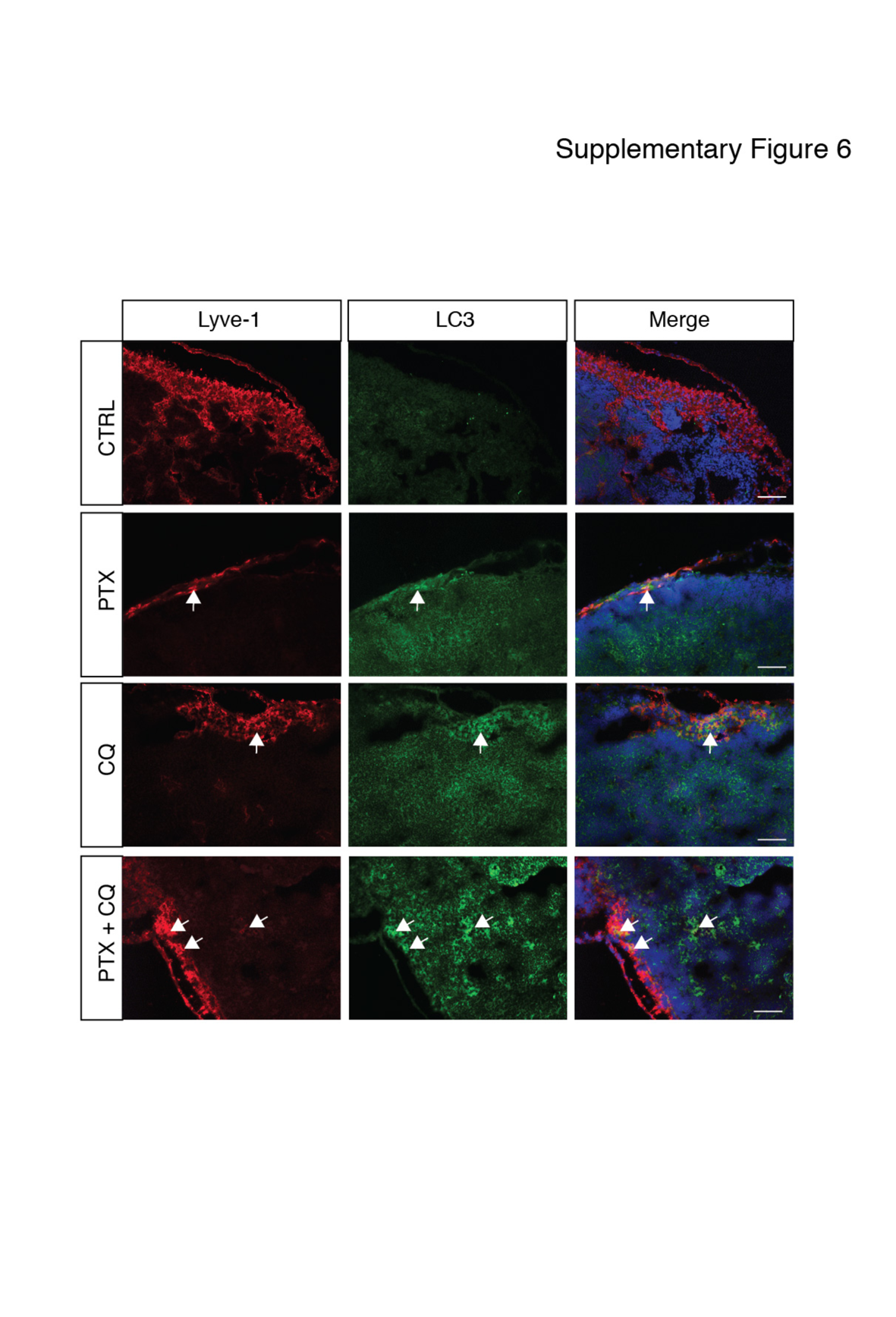

Supplement: Supplementary file 6 — Supplemental figure 6 [file 41419_2019_2181_MOESM6_ESM.tif]
