# Supplementary material for: Paclitaxel induces lymphatic endothelial cells autophagy to promote metastasis
Source: Cell Death Dis. 2019 Dec 20;10(12):956. doi: 10.1038/s41419-019-2181-1 (PMC6925245; doi:10.1038/s41419-019-2181-1)
Supplement: Supplementary file 7 — supplemental informations [file 41419_2019_2181_MOESM7_ESM.docx]

**PACLITAXEL INDUCES LYMPHATIC ENDOTHELIAL CELLS AUTOPHAGY TO PROMOTE METASTASIS**

Audrey Zamora^1^, Melinda Alves^1^, Charlotte Chollet^2^, Nicole Therville^3^, Tiffany Fougeray^4^, Florence Tatin^1^, Camille Franchet^5^, Anne Gomez-Brouchet^5^, Charlotte Vaysse^2^, Laurent O. Martinez^1^, Souad Najib^1^, Julie Guillermet-Guibert^3^, Eric Lacazette^1^, Anne-Catherine Prats^1^, Barbara Garmy-Susini^1^.

**SUPPLEMENTARY INFORMATIONS**

**Supplemental procedure.**

**Tissue Specimens**

In total, 20 primary human breast cancer specimens were collected. Specimens were obtained from archival paraffin blocks between 2002 and 2008. Ten non treated and 10 paclitaxel-neoadjuvant treated cancer patients were provided by the biology resource center of Rangueil hospital, Toulouse, France. Samples were selected as coded specimens under a protocol approved by the INSERM Institutional Review Board (DC-2008-463) and Research State Department (Ministère de la recherche, ARS, CPP2, authorization AC-2008-820) and included tumor specimens identified as invasive ductal carcinoma. All tumors were obtained from surgical resections. Final breast cancer diagnosis was confirmed by pathologic evaluation. Specimens were stained with hematoxylin and eosin (H&E) to confirm diagnoses. Each series included as controls normal breast tissue and selected common specimens to ensure consistent interpretation, as well as packed, sectioned cell lines engineered to over- or under-express specific factors.

**Reagents.**

Mouse anti-human podoplanin (clone D2/40) was from Dako. Anti-human CD3 was from Abscam.

**LC3-Tandem assay.**

Premo^TM^ autophagy Tandem sensor RFP-GFP-LC3B Kit (Thermo Fischer Scientific, Grand Island, NY, USA) was used to detect autophagic vesicles as manufacturer's recommandations. This molecular probe allowed for discrimination between autophagosomes (AP) in Green and autophagolysosomes (AL) in Red based on GFP and RFP fluorescence properties in acidic conditions. HDLEC (2x10^4^) were plated on coverslip, and grown for one day. Following overnight incubation with 10µl of Bacman reagent, the cells were pre-treated 1h with chloroquine (CQ-10 µM) and exposed in co-treatment to Paclitaxel (PTX - 10nM). The cells were fixed in PFA 4%, stained with dapi, and mounted with fluorescent mounting medium (Dako). Images were taken using confocal microscopy (Zeiss LSM 780) at a x40 objective magnification, and RFP+ spots and GFP+ spots were quantified with Image J using a filter size of 0.5 µm.
